# *TUM-ParticleTyper 2*: automated quantitative analysis of (microplastic) particles and fibers down to 1 ${\upmu }$m by Raman microspectroscopy

**DOI:** 10.1007/s00216-023-04712-9

**Published:** 2023-06-08

**Authors:** Oliver Jacob, Alejandro Ramírez-Piñero, Martin Elsner, Natalia P. Ivleva

**Affiliations:** Institute of Water Chemistry, Chair of Analytical Chemistry and Water Chemistry, Technical University of Munich, Lichtenbergstr. 4, 85748 Garching, Germany

**Keywords:** Microplastics, Morphological characterisation, Chemical identification, Adaptive de-agglomeration

## Abstract

Accurate quantification of small microplastics in environmental and food samples is a prerequisite for studying their potential hazard. Knowledge of numbers, size distributions and polymer type for particles and fibers is particularly relevant in this respect. Raman microspectroscopy can identify particles down to 1 ${\upmu }$m in diameter. Here, a fully automated procedure for quantifying microplastics across the entire defined size range is presented as the core of the new software *TUM-ParticleTyper 2*. This software implements the theoretical approaches of *random window sampling* and *on-the-fly* confidence interval estimation during ongoing measurements. It also includes improvements to image processing and fiber recognition (when compared to the previous software *TUM-ParticleTyper* for analysis of particles/fibers $> 10$ ${\upmu }$m), and a new approach to *adaptive de-agglomeration*. Repeated measurements of internally produced secondary reference microplastics were evaluated to assess the precision of the whole procedure.

**Supplementary Information:**

The online version contains supplementary material available at 10.1007/s00216-023-04712-9.

## Introduction

Most plastic materials are marketed in the form of disposables. The resulting waste is characterised by a high persistence in the environment [1–4]. Each plastic item, especially when entering aquatic ecosystems, is subject to an ongoing fragmentation, caused by physical strain, UV irradiation, and biodegradation [5]. If the size of these fragments reaches a range between 1 ${\upmu }$m and 1 mm (or 5 mm, as an alternative definition), they are called microplastics (MPs) [6–8]. Potential hazards of MPs are, for example, the physical influence on biota or their possibility to act as carrier of harmful substances. MP counts (linked to the available contact surface), size distributions (accessibility) and chemical composition are relevant properties in this context; representative and reliable results for these categories are a prerequisite to evaluate the influence of microplastic particles within this subject area.

Several methods are usable for MP analysis, which are to be divided into mass-based (e.g. thermoanalytical) and particle-based (spectroscopic) methods delivering, to an extent, complementary information [9–12].

This study addresses one type of the latter—static image analysis methods for particle size analysis (ISO 13322-1) [13] combined with Raman microspectroscopy—while focussing on the topics of image processing, object recognition and representative subsampling. In particular, we address special requirements for particles and fibers of the lower size range (1 ${\upmu }$m to 50 ${\upmu }$m, minimum Feret’s diameter), statistical methods used for that, and extensive automation as a necessary prerequisite in this context.

The typical procedure for quantification of MPs in environmental and food samples with aqueous matrices includes the filtration on a suitable substrate, e.g. Gold-coated polycarbonate membranes [14, 15] or silicon membranes [16], which means the depositing of the solid matter content on the filter membrane. Thereby it must be controlled that no significant contamination occurs from the laboratory environment or from additional preparation steps such as oxidation (e.g. Fenton reagent) to remove interferences, or density separation [17]. A typical strategy to answer the questions on fragment counts, size distribution, shape and chemical composition of MPs involves the use of commercial or in-house developed software to detect the deposited fragments in *dark-field* images and guide subsequent Raman microspectroscopic measurements on the filter. Several realisations of this approach have already been brought forward [14, 18–23]. They differ by respective lower limit of fragment sizes and represent an evolution over the years, as can be seen for the techniques used for image segmentation. Whereas Erni-Cassola et al. [19] counted on threshold values for binarisation to be selected manually—which, however, is a major drawback as results stemming from that procedure cannot be reproduced—the “Microplastics Visual Analysis Tool” (MP-VAT) [20] uses an automatically determined threshold. Going further, the use of an *adaptive threshold* as applied for *TUM-ParticleTyper* by von der Esch et al. [14] helps to take into account also local differences in illumination that would otherwise lead to erroneous detection results. The software, to give an example, enables the detection and morphological characterisation of fragments (particles and fibers) > 10 ${\upmu }$m on an entire filter surface (circular, diameter 20 mm), so that an accurate single-particle Raman measurement is subsequently possible. It also enables a validation procedure using the capability of particle detection by experts as a reference [14]. Regarding the automation of MP analysis, another open-source software, GEPARD [22], as well as commercial programmes, give the possibility to control Raman measurements and to automatically summarise the data and present the final results.

To develop the methodology in the direction of the lower limit of the size range of MPs (1 ${\upmu }$m), several challenges gain in importance, which will be discussed in the following.

As a true result for fragment counts depends primarily on a correct detection, the precision of image analysis for single-particle detection is of great importance. As a prerequisite, the image resolution has to be high enough to resolve all fragments down to the lower size limit ensuring an optical image with a sufficient number of pixels (at least 13 pixels according to von der Esch et al. [14]). Suggestions aiming at a lower minimum (e.g. 3 pixels [21]) do not seem to consider additional requirements such as those for classifying shapes. An important tool to support a correct detection is image processing carried out beforehand which can both lead to a reduction of false positive and false negative results. An example of an appropriate method is median filtering applied in *TUM-ParticleTyper* (1) [14]. The use of a more elaborate processing method, or a combination of methods, has the potential to enhance this effect and is necessary for detection of fragments in the lowest size range. Here, with a concomitant change to a higher magnification objective, features of the background (filter surface) will be more clearly visible. An (almost) extinction by median filtering is not possible anymore.

Of additional importance for a correct detection is the entire visibility of all particles after their deposition on the filter surface. A single object can be correctly detected if it is completely surrounded by (dark) background, as the detection itself is based on a threshold for brightness. Under the assumption of Complete Spatial Randomness (CSR) Xu et al. [24] showed theoretical higher limits for particle counts (including different particle sizes) if an isolation ratio of 99% has to be reached (e.g. 400 particles with a size of approx. 50 ${\upmu }$m if distributed on a filter area (314 mm$^{2}$) comparable to the one involved here). Especially when considering the need for representative sample volumes, it is not possible to completely avoid agglomeration. Since particle counts can be one to two orders of magnitude higher, any detection method will have to deal with this issue. Agglomerates lead to an underestimation of particle counts, a shift within the size classification, erroneous fiber recognition, and thus to an untrue result. The use of a de-agglomeration method appears therefore reasonable and is described, for example, by Anger et al. [23] and Brandt et al. [22]. Such a method is frequently based on watershed transformation [25]. However, this approach is prone to overfragmentation, resulting in an overestimation of particle counts, especially if many fibrous objects are involved [22, 23]. While manual correction or guidance [22] is possible, it is not reproducible and time-intensive. If an iterative automated measurement process as presented here is to be considered, it is finally impossible. Therefore, an alternative approach is necessary which accounts for the drawbacks of watershed transformation on the one hand and is fully automated on the other hand.

Another important topic is the differentiation between particles and fibers. This can be achieved by defining a morphological dimension for an object in question and applying a threshold. This dimension should ideally be independent of position and rotation of the object in relation to the coordinate system. For flexible objects, also different curvatures should not affect that value. An intuitive human recognition of fibers, which represents the reference, is certainly not based solely on a single dimension like, for example, the value 4 as a threshold for the aspect ratio given by von der Esch et al. [14]. The reason is that this change in the appearance of an object cannot be described by only one value, but requires several morphological parameters (or *shape factors*) at the same time to enable a more precise classification [26]. Typical parameters in this sense are *circularity* [20, 26, 27], *convexity* [22, 26], *elongation* or *aspect ratio* (with multiple definitions) [14, 26, 28], or *distance* [29].

The intended core function of a software for particle/fiber detection leads to the approach “one measurement point per fragment”, which is acceptable, as individual fragments are likely to consist of a material or compound in a uniform way [14, 18, 30]. This strategy is typically combined with Raman microspectroscopy, as the associated time savings are very effective compared to alternative approaches like Spectral Imaging. Since the sheer (expected) number of fragments on a filter surface does usually not allow a Raman measurement on every single object, additional strategies involve statistical methods of subsampling. A suitable approach is the random selection among all fragments as proposed by Anger et al. [30] and applied by von der Esch et al. in *TUM-ParticleTyper* (1) [14]. This approach is possible, if their positions are completely known as is the case for particles greater than 10 ${\upmu }$m. As already mentioned, measurements on particles/fibers of the lowest size range down to 1 ${\upmu }$m require special approaches for subsampling on the filter, but also to circumvent technical limitations, such as the precision of a given microscope stage. A possible solution was recently introduced in a theoretical work by Schwaferts et al. [31]. To circumvent technical limitations such as the accumulated positioning error of a microscope stage, but also to provide a basis for an approach to estimating the uncertainty of the final result for particle counts, *Random Window Subsampling* (RWS) connected to an iterative measurement scheme has been proposed, which assumes a measurement field significantly smaller than the total filter area. While the open-source software published to date, e.g. *TUM-ParticleTyper* (1), does not include any kind of error estimation for the final result, which would allow statistically sound measurements of microplastics down to 1 ${\upmu }$m, the RWS approach allows for an *on-the-fly* estimation of confidence intervals through ongoing calculations during the measurements, based on bootstrap estimates of fragment counts. Once the relative error has fallen below a predefined value, the measurement can be terminated and the final result can be calculated.

A realisation of the findings by Schwaferts et al. [31] requires a complete automatisation of the procedure, as manual steps during the iterative measurement scheme have to be avoided. This includes an exact definition of all contributing steps (like image thresholding, de-agglomeration, fiber recognition) and ensures furthermore full reproducibility of any result. Additionally, extensive automation also reduces the necessary workload of the operator to a minimum. This realisation as well as approaches towards an automatic de-agglomeration method and an advanced fiber recognition—concerning the issues raised above—is now presented as main subject of this work in the form of the software *TUM-ParticleTyper 2* as main outcome. Raman microspectroscopic measurements and their analysis by database matching, however, are aspects which are not related to the core topic of automation and object detection of this work and are therefore not discussed here. As a complete validation of the procedure is problematic since no certified reference materials exist yet, an evaluation of repetitive measurements on in-house prepared reference particles [32] was carried out instead to assess the precision to be expected involving the entire procedure (object detection, identification, and quantification of fragments).

## Materials and methods

### Generation of reference samples

The generation of secondary reference MPs was performed according to von der Esch et al. [32]. Square-cut pieces (approx. 1 mm $\times$ 1 cm $\times$ 1 cm) of three different plastic types (PS, PET, PLA) were, separated by type, subjected to ultrasonification (4 mL of 0.25 mol L$^{-1}$ KOH$_{aq}$ each, 35 kHz, 1 h), combined and filled up to 0.5 L. Aliquotation was performed according to Wolff et al. [33]: During turbulent stirring with an overhead laboratory stirrer (700 min$^{-1}=$, RZR 2000, Heidolph Instruments GmbH & Co. KG) under the presence of a flow breaker construction, two aliquots were taken through a volumetric pipette (20 mL and 60 mL) near to the stirrer blades and subsequently filtrated on Gold-coated track-etched polycarbonate membranes (pore size 0.8 ${\upmu }$m, diameter 25 mm, APC GmbH, Germany, Sartorius filtration apparatus, Sartorius AG, Germany). The filter was clamped into an according filter holder to ensure flatness of the filter as a basic requirement for the further analysis [14]. To avoid (additional) agglomeration of particles at the edges of the glass tube on top of the filter (“coffee-ring” effect), an additional layer (cellulose, circular cut out with a diameter being about 1 mm to 2 mm smaller than the one of the glass tube) is placed centred beneath the filter.

**Table 1:** Summary of the steps intended for object detection and morphological characterisation

	Step	Description
1	Adaptive Threshold$^1$	Binarisation of the original image
2	Find Contours$^1$	Boundaries around bright areas (object contours)
3	Topology$^1$	Keep the outermost contours only
4	Minimum area$^1$	Keep objects with at least 13 pixel only
5	Applicable diameter$^1$	Proceed with objects within the requested size range
*6*	*Applicable position*$^{2~3}$	Requirements for a measurement frame (for *RWS* only)
7	Shape classification$^2$	Prediction of shape category (fiber/particle)
8	Adaptive de-agglomeration$^2$	Automated separation of in-touch particles (fibers excluded)
9	Measurement points$^2$	Calculation of suitable measurement positions$^4$
*10*	*Random sampling*$^{2~3}$	Reduction of RM measurements (max. 7000, not for *RWS*)
11	Real coordinates$^1$	Calculation according to resolution (dep. on magnification)

$^{1}$(Mostly) unchanged compared to *TUM-ParticleTyper 1* [14]

$^{2}$ Optimised or entirely new parts

$^{3}$ Optional, depending on programme mode

$^{4}$ See Section “One measurement point per particle or fiber”

### Raman microscope systems

Optical image acquisition and RM measurements were performed on a WITec Raman microscope system (*alpha300*, WITec GmbH, Germany), which is directly controlled by the WITec software *Control FIVE 5.3* and *ParticleScout*. All spectra were recorded with following settings: 532 nm excitation wavelength, 3.5 mW laser power, up to $40 \times 0.5$ s integration time per particle. As the Raman measurements themselves represent the most time-consuming part of the whole procedure, they also have the greatest savings potential. *ParticleScout* offers the function “Optimize Fast”, which analyses the signal-to-noise ratio (SNR) and stops the ongoing accumulation of spectra, when the preselected SNR limit is reached or if the signal at the actual position is generally too low (Low Signal Limit). The values were set as 12 (SNR limit within a spectral range of 500 cm$^{-1}$ to 3600 cm$^{-1}$) and 25 (Low Signal Limit), respectively, after validation through experiments. The time savings can be more effective, if—starting from a fixed recording time of 20 s—a higher accumulation number and, accordingly, a lower integration time per accumulation is chosen, as this function basically needs a minimum of three accumulations before achieving any effect. The choice of this function can be reserved directly at the graphical user interface of *TUM-ParticleTyper 2*. Additionally, the inbuilt function “Spectral Auto Focus” was used (Z-axis range 10 ${\upmu }$m to 60 ${\upmu }$m, related to the z position of the filter surface, Minimum Integration Time 0.2 s, Step Size Multiplier 2.0). Although this function adds time (several seconds per particle), it helps the first-mentioned function to work more efficiently, as it is more likely to achieve a sufficient high SNR if always focusing on the surface of each particle. For database matching, WITec *TrueMatch* was used and a custom-made database was applied (including spectra of the polymer types PE, PES, PET, PLA, PMMA, polysiloxane, POM, PP, PPTA, PS, PTFE, PVC). The material revealing the highest hit quality index (Pearson correlation coefficient) after baseline correction (rolling ball algorithm [34], radius 100 pixel, considered spectral range from 590 cm$^{-1}$ to 1770 cm$^{-1}$ and from 590 cm$^{-1}$ to 1770 cm$^{-1}$) was assigned, if the value was at least 0.45. Based on the materials named above, the decision for this value is based on spectra with varying signal-to-noise ratio for measurements of both pristine and aged (known) plastic samples, so that a correct assignment, i.e. a sufficient difference to the second hit is likely. These results were automatically transferred to *TUM-ParticleTyper 2* and, in case of the RWS mode, instantly evaluated to decide whether to continue or to stop the measurement process.

As a noteworthy component of computer hardware the installed memory amounts to 64 GB, which is necessary for processing very large images and the simultaneous calculation of measurement points on particles in case of high particle loads ($> {100000}$), even though measurements will not be performed on those particles not included in a random subsample.

All measurements were directed by the automated procedure of *TUM-ParticleTyper 2*, the connection to the control software of the microscope is done through desktop recognition and simulated user interaction (Python module *PyAutoGUI*). For that reason, a set of images of all involved buttons of the graphical user interfaces was created once.

### Object detection and image processing

The core function of *TUM-ParticleTyper 2* is object detection in dark-field images. After starting the programme, the dimensions of the *field-of-view* and Raman acquisition parameters can be entered via the graphical user interface. These values are then transferred to the microscope software at the appropriate time. The reader may therefore be referred to the accompanying manual. The detection of objects on the image of a filter surface involves two functions from the *Open Source Computer Vision Library* for Python, consistent to the original version of *TUM-ParticleTyper* [14]. After the original image has been processed (filtering in the Fourier domain, top-hat transformation, morphological opening, described in Section “Image processing”) the detection of (bright) objects before a dark background is done through two functions cv2.adaptiveThreshold() (described at figure S1 in the Supplementary Material, SM) and cv2.findContours(), which are pairwise applied twice (*first* and *second run*). Useful values of the required arguments blocksize and C of cv2.adaptive Threshold() can thus be applied for both large (prevention of fragmentation) and small fragments (reduced probability for agglomeration), whereby twofold counting is naturally prevented. These parameters have a significant impact on false positive and false negative results, and detected object sizes (see Section “Object detection in dark-field images”). All objects found that way are described through lists of 2D coordinates representing their contours (one per object). If contours are enclosed by others, only the respective outermost one is further processed. Contours having pixel counts, (minimum or maximum) lengths or shapes (fiber or other), that do not match the conditions set by the user, are sorted out as well (according to steps 3 to 7, Table 1).

From the resulting set of objects, a sample for successive RM measurements is drawn (RS), if the total count of objects exceeds the intended sample length given by the user (7000 per default). Otherwise, the whole set is subjected to RM measurements. The default value results from theoretical considerations regarding the sample size dependent on the expected total particle count (e.g. 20000 particles) and, accordingly, the analyte ratio (e.g. 3%) to ensure representativeness (confidence level 90%) [30]. By that, the typical duration of the whole measurement procedure can be limited (e.g. about 2d when an acquisition time of 20 s per fragment is set). All spots to be measured are drawn into a black and white image of same size as the original image as the transfer of the spot coordinates into WITec *ParticleScout* can only be achieved graphically. The latter programme then conducts all Raman measurements using the parameters previously entered into *TUM-ParticleTyper 2* (refer to the manual). This, the successive database analysis, and export of results are initialised by *ParticleTyper* through simulated user interaction.

### Validation of particle recognition

To assess the precision of the procedure based on RWS, ten repeated measurements were performed on the same sample (20 mL aliquot) and with the same settings as follows: The positions for 100 randomly placed windows (70 ${\upmu }$m $\times$ 70 ${\upmu }$m) were determined, fragments from 1 ${\upmu }$m to 50 ${\upmu }$m were to be considered (objective $100 \times$, N.A. 0.9, working distance 1 mm, Carl Zeiss AG, Germany) and the according field-of-view was 120 ${\upmu }$m $\times$ 120 $\upmu$m. From the results of object detection, point measurements (one per object) were performed at the calculated spot positions. At each window position, the sequence of image acquisition and processing, object detection, Raman analysis (*alpha300*, WITec) including database matching, and final data preparation was automatically processed and repeated at all remaining window positions. After every tenth window, a bootstrap-based confidence interval was calculated (confidence level 90%) including the data of all windows measured until then. After all windows were processed, the final result was calculated.

To assess the effect of higher probability of agglomeration on the filter due to an increased particle load, five repeated measurements were each performed on two samples (20 mL and 60 mL aliquot) and with the settings as follows (RS mode): A circular image (diameter 20 mm, resolution 0.873 ${\upmu }$m) was taken and the sequence (image processing, object detection, point measurements, final data preparation) as described above was performed while taking fragments from 10 ${\upmu }$m to 1000 ${\upmu }$m into account.

## Results and discussion

### TUM-ParticleTyper 2

**Fig. 1:**
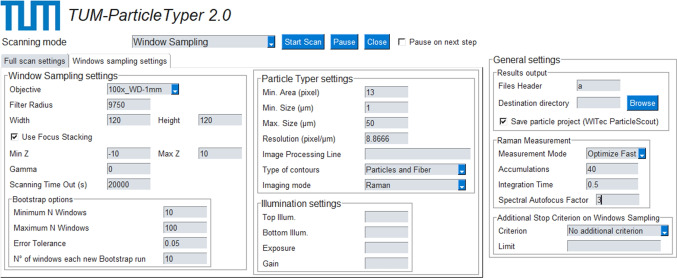
Screenshot of the graphical user interface of the software *TUM-ParticleTyper 2*

*TUM-ParticleTyper 2* (Fig. 1) provides full automation of all analysis steps starting after sample placement beneath an objective of the Raman microscope and resulting in material dependent calculations of histograms of particle/fiber size distributions. Any subjective bias can therefore be prevented. Based on desktop recognition and simulated user interaction, the software maximises the functionality of a Raman system for an optimal detection, quantification and morphological characterisation of (plastic) microparticles and fibers.

When compared to *TUM-ParticleTyper 1* (suitable for characterisation of particles/fibers down to 10 ${\upmu }$m) [14], the software presented here enables now the representative analysis of fragments down to 1 ${\upmu }$m (minimum Feret’s diameter).

This is mainly reached by significant improvements in image processing and the full implementation of the *random window sampling* approach (RWS) proposed by Schwaferts et al. [31]. In addition, new approaches for improved fiber detection (Section “Shape classification”) and a fully automated way for appropriate separation of in-touch fragments (Section “Adaptive de-agglomeration”) are included.

Finally, an experimental validation of RWS provides information about the precision of the entire automated analysis.

**Fig. 2:**
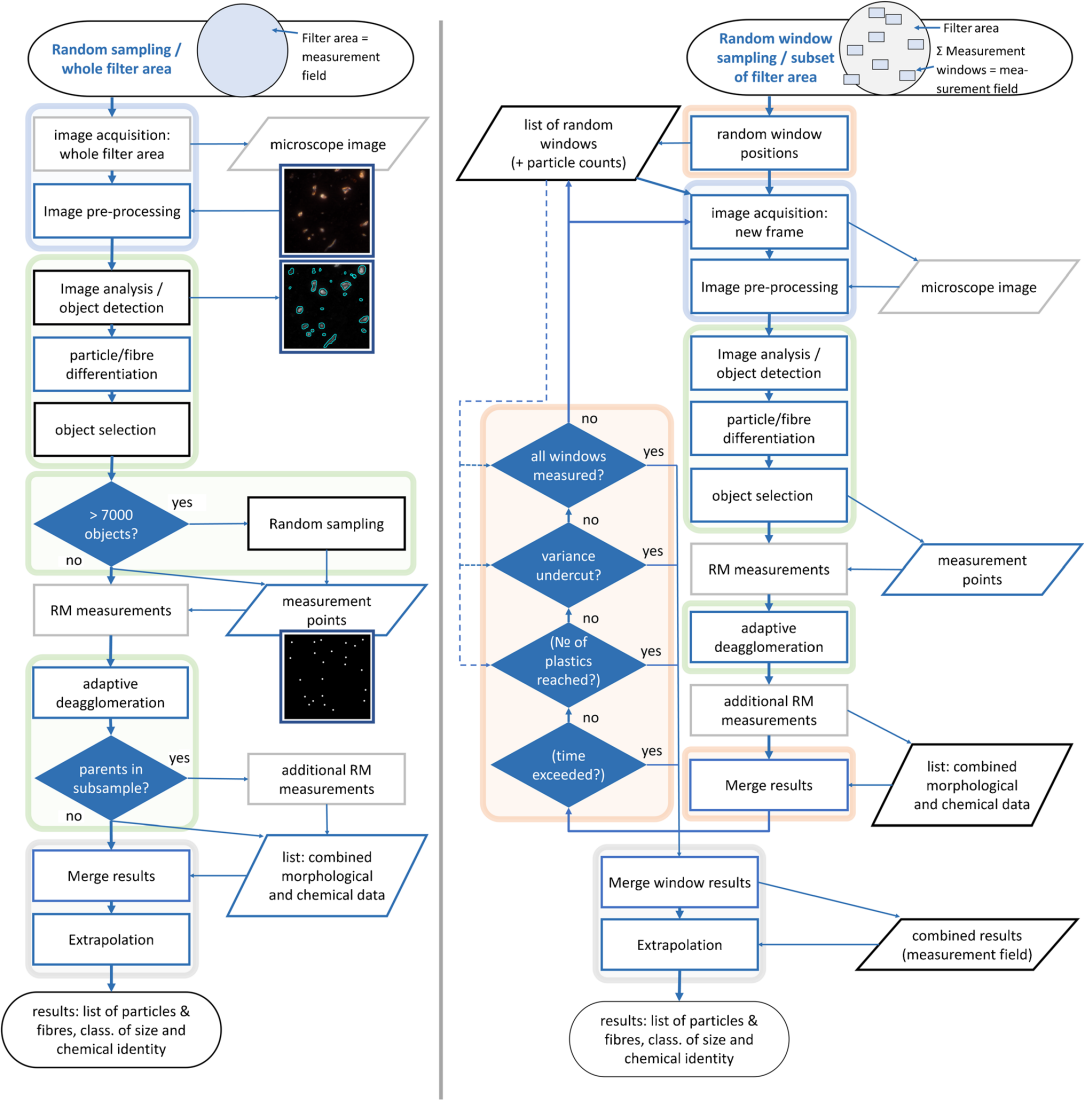
Flow chart depicting processes during the analysis by *TUM-ParticleTyper 2*. Left side: *Random sampling* (for analysis of fragments sized $\le 10$ ${\upmu }$m. Right: *Random window sampling* (for fragments sized $\le 1$ ${\upmu }$m). *Rectangles* denote processes, *parallelograms* data input and output, and *rhombuses* decisions. When compared to *TUM-ParticleTyper 1* [14], a blue colour of borderings stands for new or further developed functions (grey borderings denote steps not controlled but only activated by *ParticleTyper*), which is also true for all decision steps. Coloured background marks groups which are discussed in coherent Sections: blue in Section “Image processing”, green (image analysis) starting from Section “Object detection in dark-field images” up to Section “Adaptive de-agglomeration”. Red background refers to automation and window related steps (RWS mode). Fields highlighted in grey belong to the Section “Evaluation and presentation of results”

There are two subsampling methods available in *TUM-ParticleTyper 2* to shorten the measurement time: *random sampling* (RS) of particles for the analysis of fragments $> 10$ ${\upmu }$m and RWS for fragments within the size range 1 ${\upmu }$m to 50 ${\upmu }$m; two methods can also be applied in sequence. RS requires objectives with lower magnification (e.g. $20 \times$). Here, fragments to be measured are randomly selected after their detection on the entire filter surface [14, 17, 30]. RWS (designed for the use of higher magnification objectives (e.g. $100 \times$, $50 \times$)) moreover allows variance estimation *on-the-fly* based on bootstrap samples and an automatic termination of the window measurement loop [31]. Here, measurement windows are randomly placed over the whole filter area, where particle detection followed by RM measurements is pairwise sequentially conducted, until predefined criteria are met (maximum number of plastic particles/fibers to be measured or maximum measurement time reached, maximum number of windows reached, given uncertainty of result undercut). Finally, fragment counts are extrapolated according to the achieved window subset area proportion (RWS) or to the counts of all detected (but not necessarily measured) fragments per size class. An overview for both approaches (RS and RWS) is given by Fig. 2.

### Image processing

Three distinct methods of image processing are here applied that aim for a more precise and correct object detection, in particular, *filtering in the Fourier domain* and *top-hat transform*, followed by *morphological opening*. A general overview about these methods can, inter alia, be found in “Computer and Machine Vision: Theory, Algorithms, Practicalities” by E. R. Davies [35]. If the entire surface of the filter is to be depicted (for RS), a circular mask is additionally applied beforehand to the image to erase the parts of the filter holder [14] that would otherwise appear at the edges of the image. For all output of processed images, the dimensions of the original image are preserved which is crucial for an unambiguous link of positional statements during the procedure. For RWS, the dimensions of the image (describing the *field-of-view*) exceed the one of a single window (denoted as *measurement frame*), in accordance to the recommendations of the International Standard 13322, part 1 (2014) [13].

*Filtering in the Fourier domain*, which enables background reduction, is the first and crucial step in image processing. The original image is therefore divided into *N* sections of same size. From every section, a new image *s* is created by mirroring and stitching until the original dimensions are rebuilt. All these mirrored images $s_i$ are analysed by the Fourier transform *F* and then multiplied (Eq. 1).

1 $$\begin{aligned} H = \prod _i F_{s_i} i = 1,2,3 \dots N \end{aligned}$$

The resulting Fourier filter *H*, representing the spectrum of spatial frequencies, shows higher intensities when accordingly sized similar objects are frequently present, which is true for characteristics of the filter surface and especially for the filter pores as they are present in the whole original image. These objects are then located in a similar region of the Fourier spectrum and can therefore be attenuated effectively, whereas particulate objects stemming from the sample stand out and therefore remain almost untouched. Subsequently, the image is recreated by the inverse Fourier transform from the modified spatial spectrum $F_{\text {new}}$ according to Eq. 2.

2 $$\begin{aligned} F_{\text {new}} = F_{\text {original}} \cdot \frac{F_{\text {original}}}{F_{\text {original}} + H} \end{aligned}$$

**Fig. 3:**
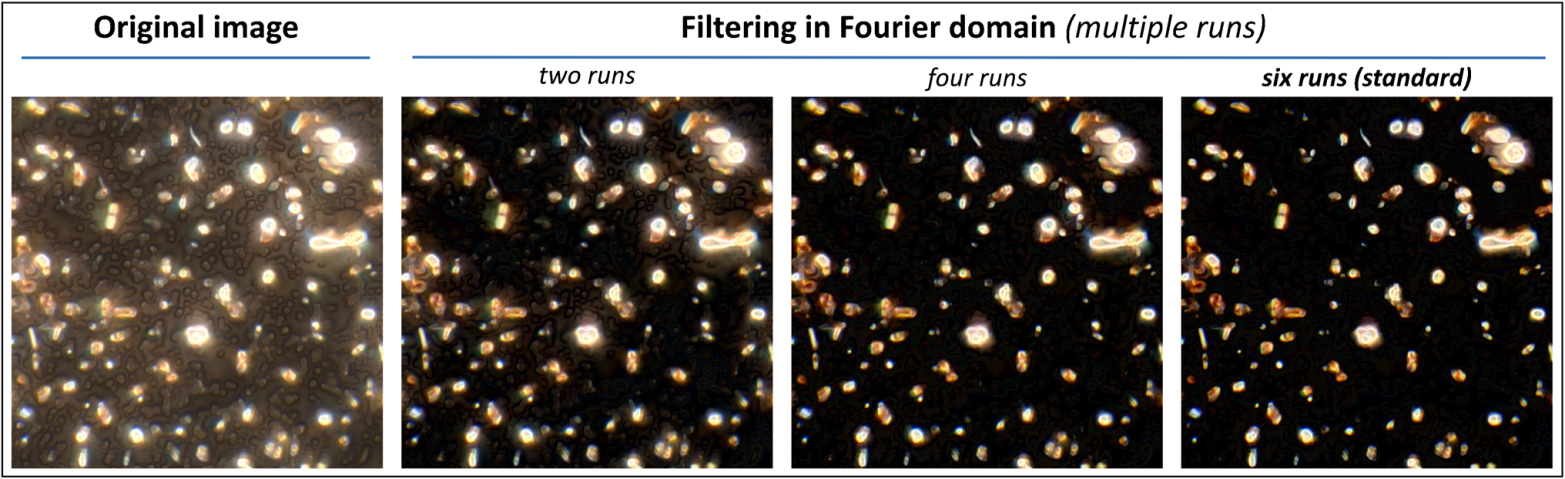
Illustration of filtering in the Fourier domain. Repeated application leads to a more effective suppression of background features while remaining object features almost untouched

This process of background reduction is repeated several times, leading to an even more effective accentuation as a gradual reduction of interfering background features can be observed (Fig. 3). Further advantages are, on the one hand, the preservation of sharpness of object contours and, on the other hand, a scaling invariant method, which means that there is no discrimination in the way particles of different sizes are processed.

The second method for image processing, the *top-hat* algorithm, specifically improves the recognition of small particles in the neighbourhood of bigger and high reflective particles (see SM, figure S2). However, size and shape of the latter might also be distorted which is a drawback of the method. In view of the reduction in the rate of false negatives, this may still be acceptable.

The third method, *morphological opening* (on grey values), can lead to a removal of small features which are below the demanded minimum size, but still have an influence on object detection leading to false positive results. In particular, the effect of *median blur* used by *TUM-ParticleTyper 1*—for its advantages see von der Esch et al. [14]—is already integrated. *Opening* consists of morphological erosion (of bright pixels) followed by dilation until the original object dimensions are restored. Objects being smaller than the range of erosion in step 1 will disappear and thus not be recoverable through dilation.

Compared to the sole application of a median filter [14], the processing steps presented here result into an improved starting point and, thus, performance during object detection.

**Fig. 4:**
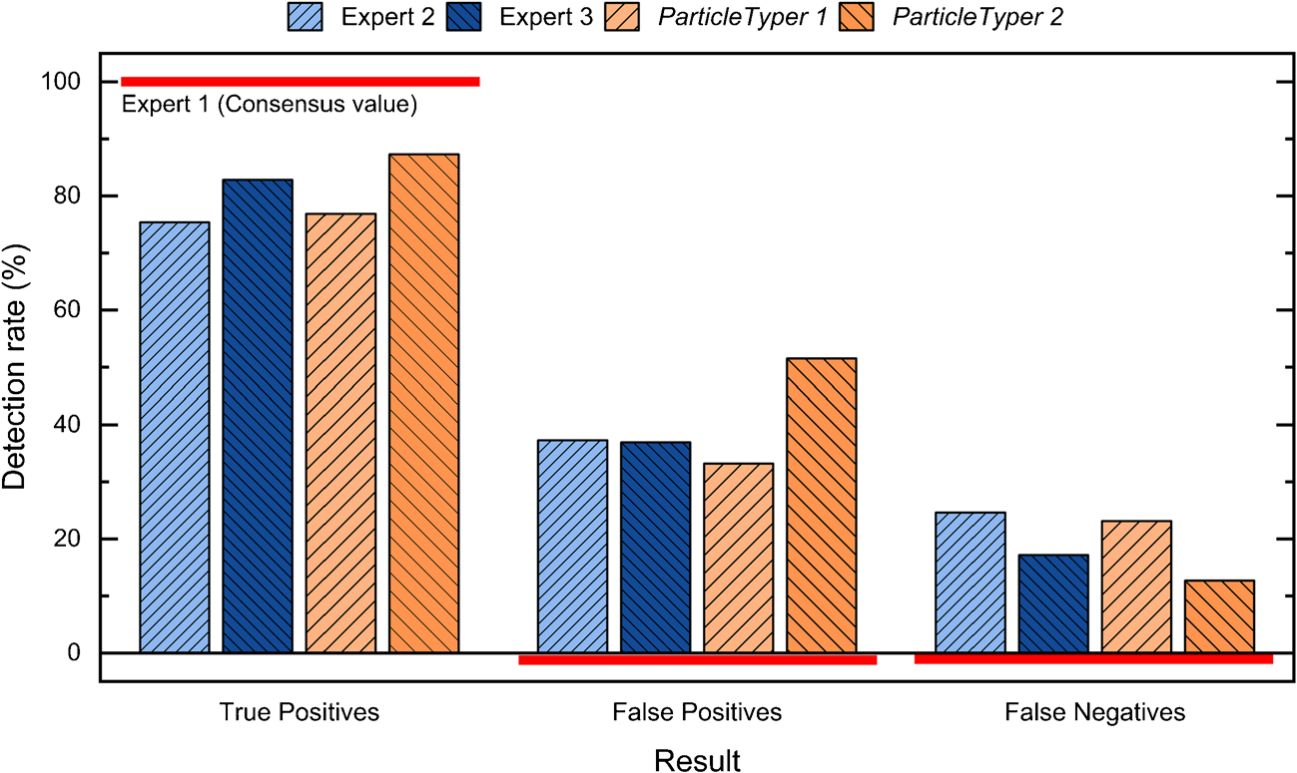
Comparison of average detection rates between three experts, *TUM-ParticleTyper* (version 1) [14] and *TUM-ParticleTyper 2* (this work) with Expert 1 being set as reference

### Object detection in dark-field images

Appropriate settings for the arguments blocksize and C of the function cv2.adaptiveThreshold() have been found and validated by von der Esch et al. [14]. Values were determined to $-6$ (C), 243 (blocksize, *first run*), and 101 (blocksize, *second run*). The blocksize at *second run* has been slightly modified to improve contour detection (91 instead of 101). Small changes of this value have no significant influence on resulting particle counts. However, if chosen too low, there is an increasing probability for artificial fragmentation. In this case, the assessment by an expert serves as criterion. When compared to von der Esch et al. [14], the values for blocksize are transformed into a representation that takes advantage of the new ability of the microscope software (WITec *Control FIVE* and WITec *ParticleScout*) to export stitched images at a constant resolution, regardless of physical size. The values are applied in this way for *ParticleTyper 2*.

Found objects are consecutively filtered according to several criteria (outermost contours, minimum area $> 12$ pixel, minimum Feret’s diameter above a minimum, maximum Feret’s diameter below a maximum required value). Where most of the criteria and their thresholds are consistent to the work of von der Esch et al. [14], the relevant value for particle size classification is now given by the minimum (rather than the maximum) Feret’s diameter; nevertheless, both diameters (as well as the area) are stored as part of the final result.

Thereafter, the new approaches for a classification of found objects into particles and fibers and *adaptive de-agglomeration* are applied. Another noteworthy feature is the improved method shown here for determining the measuring points for the Raman analysis. All steps regarding to the detection and handling of objects are summarised in Table 1.

For validation of the results of object detection, six images depicting fragments under *dark-field* illumination and taken under equivalent conditions were manually analysed by three experts (as the human recognition ability is generally accepted as “gold standard” within this context). As already demonstrated by von der Esch et al. [14] the result of object detection performed by *TUM-ParticleTyper 1* is comparable to the results of two experts. In this study the same approach was chosen to show also the effects of an improved image processing while additionally involving the results of a third expert (Fig. 4). The result of *ParticleTyper* is said to be valid, if the difference in recognition between two experts exceeds the difference between one expert and *ParticleTyper*. The new programme version shows improved detection rates with the True Positive rate being now closer to one expert as it is the case for two other experts. This is possible because *ParticleTyper 2* finds more objects overall, which leads to a higher rate of false positives. Yet this does not inevitably mean a high number of random contours but rather a higher (and consistent) sensitivity towards particularly faint objects which could be overlooked by the experts. Moreover, fluctuations can be expected for those objects with its size as seen by an expert (and painted accordingly) being smaller than detected by *ParticleTyper* if this occurs in the range of the threshold area (13 pixels).

### One measurement point per particle or fiber

Suitable measurement points are then calculated for all selected objects. Whereas this seems to be trivial for convex and more or less circular shaped contours, fibrous and other elongated or curved objects lead to useless results if the measurement point was placed at the centroid. In *ParticleTyper 1* an algorithm is applied for the latter cases which randomly moves the intended spot in different directions until it lies within the contour. However, this often results in a remaining misplacement after the maximum number of random steps has been reached, or when the last random movement ends close to the edge of the particle, so that the confocal volume is only to a small extent filled by the sample during the Raman measurement.

**Fig. 5:**
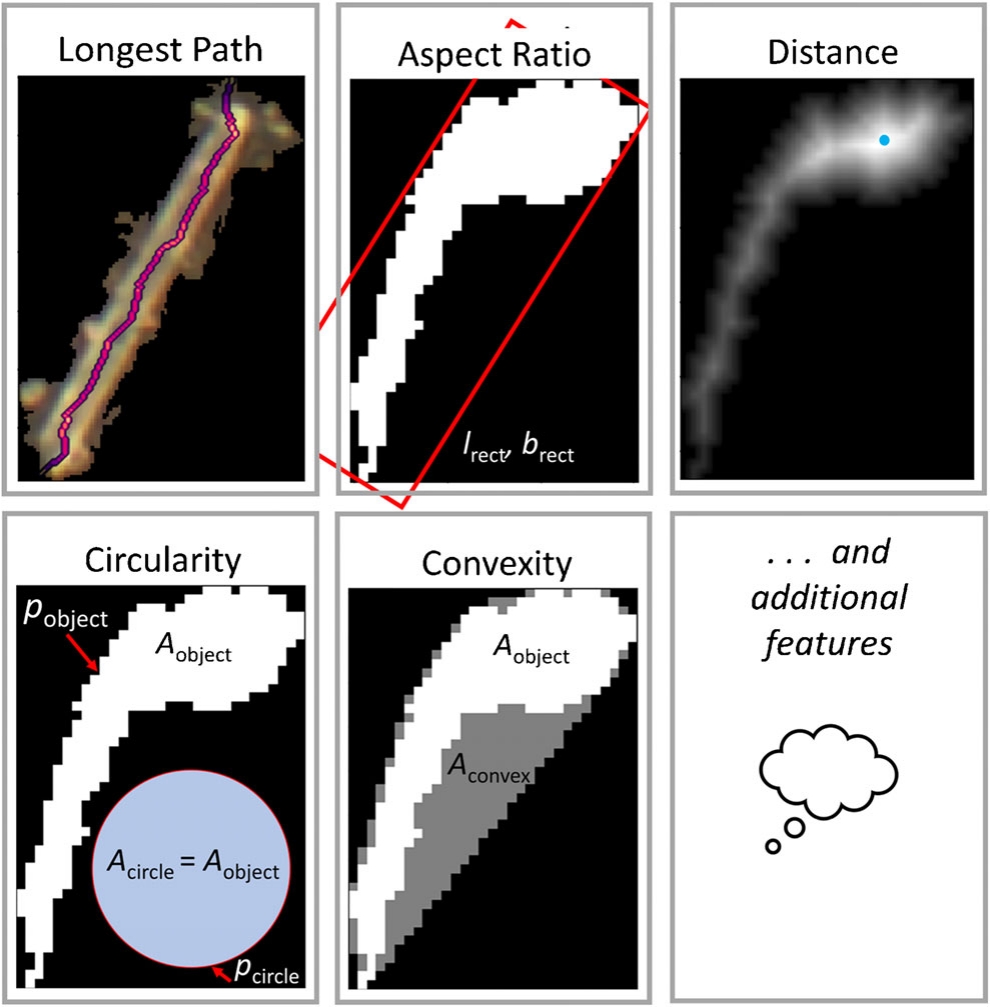
Graphical description of morphological parameters taken from each detected object. Additional useful features can be seamlessly integrated into this approach as it is generally meant as new suggestion to the topic of fiber recognition

*ParticleTyper 2* uses a new function instead which takes the object brightness into account. At first, an image section showing only the object in question is eroded and multiplied by its distance transform (a pixel within the contour gets the brighter the more it is afar from any background pixel). The brightest pixel resulting from these operations is then chosen as measurement spot. As an example, the resulting measurement point for the object shown in Fig. 5 is highlighted there with a blue dot (distance transform, top right). This procedure ensures a spot being located at a position where the object tends to be widest and at the same time set not in proximity to its border. Incorrect positioning is therefore almost impossible.

We note that the evaluation of the precision reached for the analysis of small MP fragments (1 ${\upmu }$m to 50 ${\upmu }$m, Section “Experimental validation of the RWS concept”) does naturally include the chemical identification of fragments as an integral part of the whole method for MP analysis.

### Shape classification

Shape classification is done by applying a random forest classifier model involving a set of values which are derived from several shape descriptors rather than using a threshold value for a single shape descriptor like the aspect ratio.

To achieve an automated differentiation between microfibers and other fragments, a sorting of detected objects is usually accomplished by defining a threshold for a specific parameter to describe its shape. An example is the aspect ratio, which can be expected to be similar for most fibers. However, a comprehensible threshold value is rather unclear, as already mentioned in the introduction. To answer the crucial question of “What is a fiber?”, a new approach is presented here, which takes at first the decision of an expert towards this issue into account. To this end, a reference set was first created in that sense, which consists of 1861 objects, from which 225 were classified as fibers by manual colouring in a filter image stemming from a sample of washing machine water. As suggested by Ilic et al. [26], not only one, but six different features per contour ($f_1$ to $f_6$, Eqs. 3 to 8) are calculated from that reference set. These are derived from five morphological parameters shown in Fig. 5. These six features are then used as input for a random forest classifier. The model generated in this way is subsequently applied to all object contours found by the previous steps of object detection. All necessary files for recreation, adaption or further improvement of the model created in this way are made available together with *TUM-ParticleTyper 2* itself.

3 $$\begin{aligned} f_1&= \frac{p_{\text {circle}}}{p_{\text {obj}}} \text {, with } a_{\text {obj}} = a_{\text {circle}} \end{aligned}$$

4 $$\begin{aligned} f_2&= \frac{a_{\text {obj}}}{d_{\text {Feret, min}} \cdot d_{\text {Feret, max}}} \end{aligned}$$

5 $$\begin{aligned} f_3&= \frac{d_{\text {Feret, min}}}{d_{\text {Feret, max}}} \end{aligned}$$

6 $$\begin{aligned} f_4&= \frac{a_{\text {obj}}}{a_{\text {obj, convex}}} \end{aligned}$$

7 $$\begin{aligned} f_5&= \frac{\sqrt{a_{\text {obj}}}}{d_{\text {Feret, max}}} \end{aligned}$$

8 $$\begin{aligned} f_6&= \frac{\text {SD}(d_{\text {l.br.}})}{\bar{d}_{\text {l.br.}}} \end{aligned}$$

**Fig. 6:**
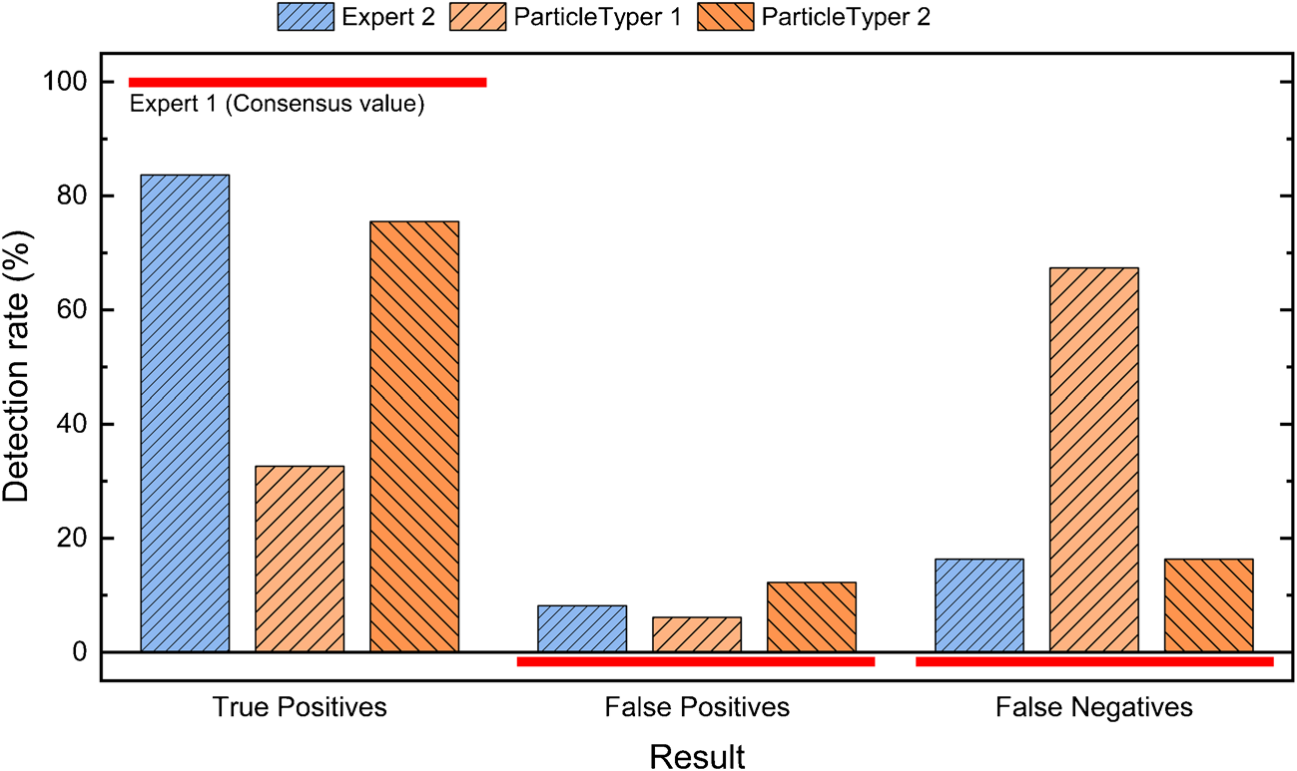
Comparison of results of fiber recognition between two experts, *TUM-ParticleTyper* (version 1) [14] and *TUM-ParticleTyper 2* (this work) with Expert 1 being set as reference

As long as a fiber is placed flatly on the surface, its length is correctly stated by the maximum length of the skeletal branches instead of the maximum Feret’s diameter which will give too small values if the fiber is curved. The skeleton of a contour is calculated by the functions medskel() and analyze_skeletons() which are part of the Python library filfinder [36]. Since the calculation may not work correctly in all cases, the maximum Feret’s diameter is then automatically used as the indication for the fiber length.

Figure 6 shows the evaluation of fiber detection in a similar way as it was done for object detection (Table 1). Referred to the classification by an expert, the method presented here leads to an improved recognition of fibers especially in the case of curved positions.

As a suggestion for future improvements, additional suitable parameters/features leading to an even more precise classification can simply be added.

### Adaptive de-agglomeration

**Fig. 7:**
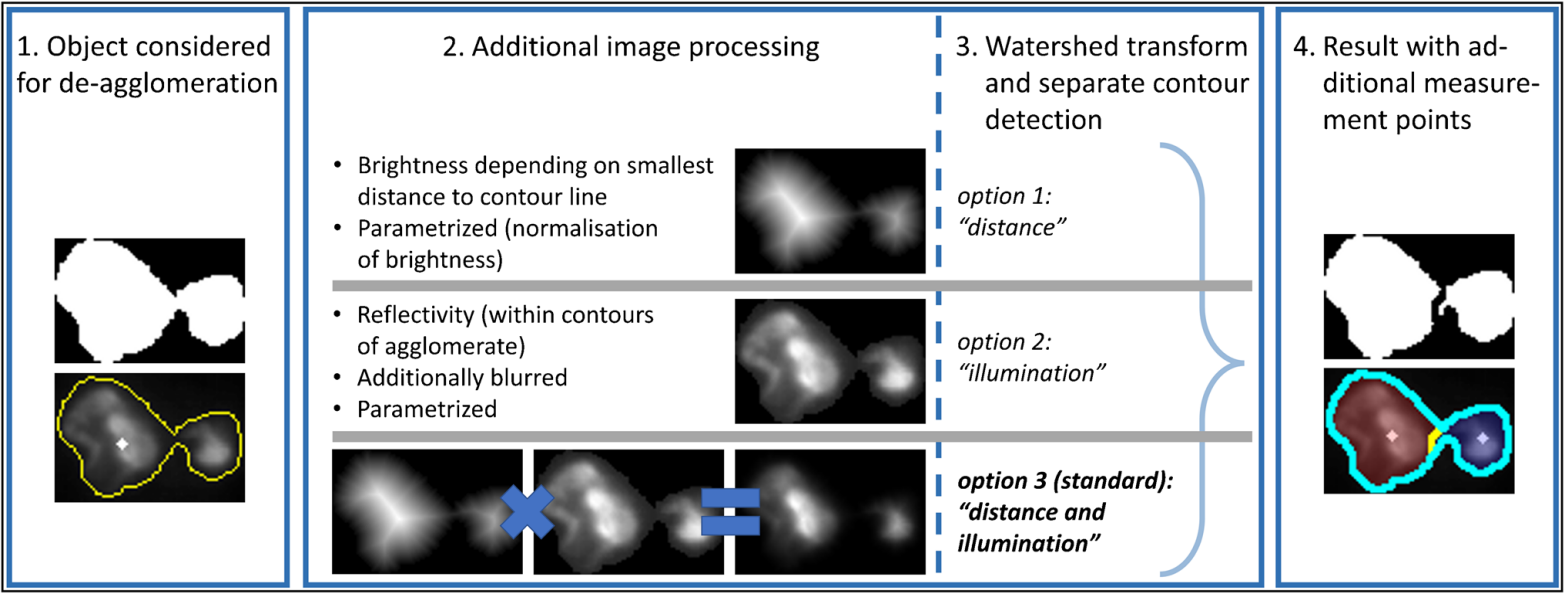
Description of *adaptive de-agglomeration* as a new approach presented in this work. Steps 2 and 3 (additional image processing, watershed transform) are applied only to objects which were considered for de-agglomeration beforehand (step 1)

Here, a new method is presented that intends to combine a de-agglomeration method involving watershed transform in a way being not prone to overfragmentation on the one hand, and to generally avoid its application on fibers on the other hand. Additionally, only (remaining) objects possessing a distinct concave shape are considered for de-agglomeration. In preparation, all objects are manually classified into (singular) particles and agglomerates once in a typical *dark-field* image. The two groups can then be separated according to the distribution of convexity values using Otsu’s algorithm, which is a well-known method for automatic threshold selection [37]. For all following images, only those (non-fibrous) objects surpassing this threshold of convexity are considered for de-agglomeration. The chosen objects are then further processed. Per default, the masked image of a single object is eroded and multiplied by its distance transform (“option 3” in Fig. 7). Being prepared that way, it is subjected to watershed transform, followed by additional contour detection and calculation of additional measurement spots. A division of parent objects is not necessarily the case, of course. Only if at least two children per parent object are generated this way, they will be represented within the final result (output files final_result_size_classification⁎.csv) in- stead of the former parent particle. However, the final list of single particles (final_result_all_particles. csv)^1^ still contains those parents with their determined parameters and chemical properties. A graphical description of the whole procedure is presented again in Fig. 7.

When looking at the effect of this method on object detection in a section of a typical filter image, an object count without additional de-agglomeration leads to a particle count of 258. Manual counting by an expert, on the other hand, reveals at least 361 objects. Including *adaptive de-agglomeration*, the count after automated detection rises to 344 corresponding to an improved trueness of object detection while at the same time being fully reproducible as no manual intervention meaning an introduction of human bias is further required.

**Fig. 8:**
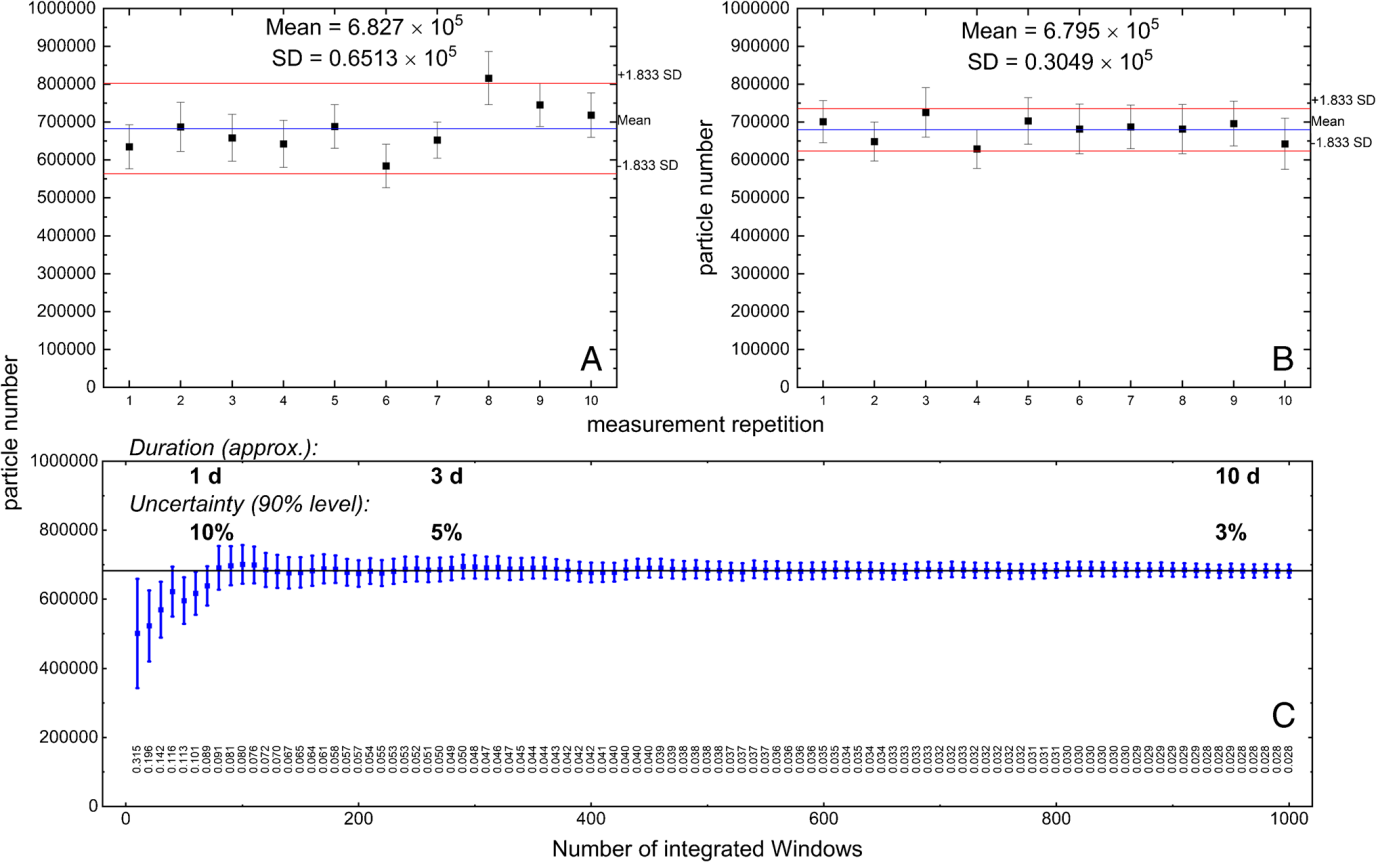
Results of ten repetitive measurements (RWS mode) on microplastic particles $> 1$ ${\upmu }$m (PS, PET, PLA) generated by ultrasonic treatment (Materials and Methods) (**A**), results of ten virtual measurements (regrouping of 1000 single windows in random order) (**B**), and progression of the bootstrap-based confidence interval [31] (1000 single windows in random order, now representing one coherent measurement) (**C**)

**Fig. 9:**
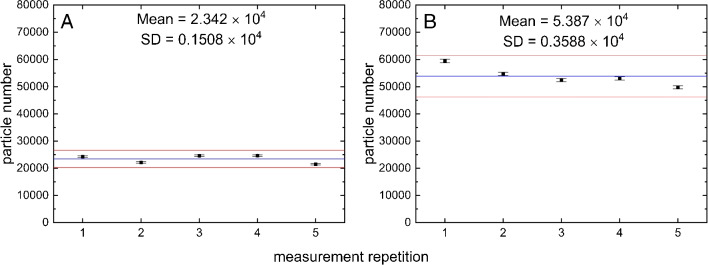
Results of five repetitive measurements (RS mode) on MP fragments $> 10$ ${\upmu }$m (PS, PET, PLA) generated by ultrasonic treatment after filtration of a 20 mL aliquot (**A**) and a 60 mL aliquot (**B**)

### Evaluation and presentation of results

Results of object detection (output by *TUM-ParticleTyper 2* in tabular form) and material identification by RM measurements (tabular output by WITec *ParticleScout*) are merged according to measurement spot coordinates. After the object classification (fiber recognition) and, if applicable, extrapolation according to the chosen method of subsampling has finished, these results are automatically evaluated and presented in tabular (list of all detected particles, list of particle counts per size class and material) and visual form (microscope images in original and preprocessed form, additionally overlaid by recognised contours distinguishable drawn according to their classification).

### Experimental validation of the RWS concept

To validate the concept of random window sampling and the bootstrap-based calculation of confidence intervals, the results from repeated measurements of reference particles are evaluated for variance. Ten consecutive measurements under equal conditions on the same sample result in 5.776 $\times$ 10$^{5}$ to $\times$ 8.125 $\times$ 10$^{5}$ plastic particles (almost exclusively consisting of PS, PET, PLA) applying the whole measurement procedure involving *random window sampling* offered by *TUM-ParticleTyper 2*. Each result consists of the measurements on 100 randomly placed windows (detailed results of these measurements are given in the SM, figures S3 to S12). As this procedure can also be seen as a series of 1000 windows measured one after another under equal conditions, it can be concluded that there should be a negligible difference in uncertainty when observing arbitrary groups of same length (100). Accordingly, the blue error bars which denote the remaining uncertainty estimated from bootstrap samples (90% level) are of similar size (top left and right diagrams). However, when estimated through standard deviation (SD) from the ten final results (real order of $10 \times 100$ windows) as seen in Fig. 8 (top left diagram, three outliers out of ten results according to bootstrap estimates), the uncertainty is about twice as high as estimated from bootstrap samples. Of two possible reasons—erroneous bootstrap estimation or altered measurement conditions—the first one can be excluded since after regrouping the 1000 underlying window results in a random manner there is an approximate consistency between the two methods for estimating the uncertainty (top right diagram, one outlier out of ten results). Consequently, there must be an additional factor of between-group variance that can therefore not be captured by estimation through bootstrap samples. As already shown theoretically by Schwaferts et al. [31] the longer the measurements are going on in terms of overall number of windows, the more precise a result can be. Lengthy and time-consuming measurements are often not practicable; this method can provide results within a few hours at the cost of higher uncertainty as shown in Fig. 8 (bottom). With the given instrumentation the remaining uncertainty presented here (about 5% to 8% at 90% confidence level) was reached after 1d of measurement time in each case.

### Influence of particle density on the result

Figure 9 shows two diagrams resulting from measurements on two samples of reference particles based on two different aliquot volumes (20 mL, **A**, and 60 mL, **B**). Random sampling was chosen for automatic measurements corresponding to a lower size limit of 10 ${\upmu }$m (minimum Feret’s diameter) and thus a focus on bigger particles (up to 1000 ${\upmu }$m). The measurements result in 2.342 $\times$ 10$^{4}$ particles (**A**) and 5.387 $\times$ 10$^{4}$particles (**B**), respectively. Compared to Fig. 8 it is first noticeable that the error bars are smaller by about one order of magnitude related to the uncertainty derived from the standard deviation of the single results. This is due to the exclusive reference on the random choice of particles when a certain ratio of plastic and non-plastic particles of the population is given. However, the inclusion of Raman-based information on the chemical identity of each particle is not considered here. A calculation based on the real variability in this sense, as it is the case for random window sampling, is therefore not possible. This estimate of uncertainty can thus only be considered as the smallest possible error that can be made using *Random Sampling* and depending on the chosen sample length (7000 per default).

A second peculiarity relates to the variation in particle counts depending on the underlying aliquot volumes. Under the assumption that the subsampling works properly, it can be seen that a tripling of the original sampling volume (20 mL) and thus a corresponding increase in the expected particle load does not lead to a tripling of the resulting particle counts after the measurements. This has to be expected because of the higher probability of overlapping when a higher amount of particles is deposited on a constant area so that an increasingly untrue result has to be expected. A theoretical description to this is, inter alia, given by Xu et al. [24]. The *adaptive de-agglomeration* method described here can, to a certain extent, migitate, but not eliminate, these effects. As a consequence, the use of an aliquotation method and the determination of an appropriate aliquot volume is of great importance as it does not necessarily lead to a more precise (due to an additional uncertainty of the aliquotation itself), but possibly to a particle count result of greater trueness.

When real samples are to be measured, where a very high number of non-plastic particles has to be expected—even if a suitable sample preparation was been applied before—this aspect gets relevant. In general, interference from these matrix particles is less likely as long as the particles are mostly separated and plastic particles are not covered, leading to false negative results otherwise. However, a lower ratio of plastic to non-plastic particles results in a prolonged measurement time if a similar relative error is to be achieved.

## Summary

For MP analysis, a combination of recognition in optical images followed by Raman microspectroscopic measurements is employed. When advancing this methodology towards the lower limit of the defined size range ($> 1$ ${\upmu }$m), aspects like representativeness but also technical restrictions of the instrument (i.e. precision of the microscope stage) become increasingly important. As a solution, a measurement scheme based on *random window sampling* can be applied which gives additionally the possibility for a bootstrap-based estimation of the remaining uncertainty, after a certain number of particles/fibers was measured, and to resume those measurements, if a predefined precision value has not been reached so far. This approach was theoretically described by Schwaferts et al. [31] and is now fully realised as part of the newly developed software *TUM-ParticleTyper 2*. Based on the recently published work of von der Esch et al. [14] the software offers the whole functionality of the original programme, i.e. object recognition in *dark-field* images or fluorescence images, *random sampling* of objects to be measured, and the calculation of suitable measurement points for subsequent Raman analysis. This functionality, however, is now embedded into a fully automated measurement process starting after sample placement beneath the microscope objective and finishing with a presentation of the final result including extrapolation and size classification for differentiable plastic material types. The software consists of an extended procedure for image processing (containing *filtering in the Fourier domain*, *top-hat algorithm*, and *morphological opening*) which aims for the reduction of false positive and especially false negative object detection results. This is followed by a new approach for an automated *adaptive de-agglomeration*, as the general application of this type of post processing seems to be inevitable, as otherwise it is very likely to risk a significant underestimation of fragment counts. Another type of post processing refers to differentiation between particles and fibers. Along with *TUM-ParticleTyper 2*, a model for fiber recognition is provided, which is derived from the according classification of an expert and which takes five morphological parameters into account. Its application leads to a detection rate of 80% when compared to the results of an expert. Overall, results for classification of fragments in particles and fibers, counts, minimum and maximum Feret’s diameters as well as the areas are given. Additionally, values for morphological parameters used for classification are also included. The new ability of this programme, the estimation of uncertainty based on a single result when applying *random window sampling*, was tested by repetitive measurements on the same sample containing secondary reference MPs which revealed an additional statistical variation through an external influence while at the same time—through virtual mixing of single window results—the correctness of the bootstrap-based estimation of uncertainty could be approved.

In summary, the integration of optimised image processing, random window subsampling and bootstrap-based confidence intervals, combined with extensive automation, enables representative and quantitative chemical analysis of (microplastic) particles and fibers down to a diameter of 1 ${\upmu }$m.

## Supplementary Information

Below is the link to the electronic supplementary material.

Supplementary file 1 (pdf 0 KB)

## Data Availability

The Python code is available under https://mediatum.ub.tum.de/1707179.

## Footnotes

1 A detailed description of all output files is given in the *ParticleTyper* manual.
