# Supplementary material for: TUM-ParticleTyper 2: automated quantitative analysis of (microplastic) particles and fibers down to 1 μm by Raman microspectroscopy
Source: Anal Bioanal Chem. 2023 Jun 8;415(15):2947–61. doi: 10.1007/s00216-023-04712-9 (PMC10284940; doi:10.1007/s00216-023-04712-9)
Supplement: Supplementary file 1 — (pdf 0 KB) [file 216_2023_4712_MOESM1_ESM.pdf]

TUM-ParticleTyper 2: Automated Quantitative Analysis of (Microplastic) Particles and Fibers down to 1 µm by Raman Microspectroscopy

Oliver Jacob[1†], Alejandro Ramírez-Piñeiro[1†], Martin Elsner[1] and Natalia P. Ivleva[1*]

[1*]Institute of Water Chemistry, Chair of Analytical Chemistry and Water Chemistry, Technical University of Munich, Lichtenbergstr. 4, Garching, 85748, DE.

*Corresponding author(s). E-mail(s): natalia.ivleva@tum.de; Contributing authors: oliver.jacob@tum.de; ramirezp@in.tum.de; m.elsner@tum.de;
†These authors contributed equally to this work.

Supplementary Material (SM)

S.1 Illustration of adaptive thresholding

Figure S1 illustrates the principle of the Python function `cv2.adaptiveThreshold()` as used by *TUM-ParticleTyper 1* [1] and *TUM-ParticleTyper 2* (this work).

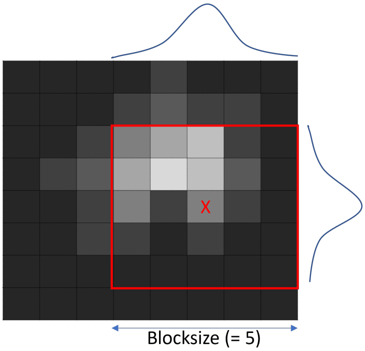

Fig. S1 Principle of binarisation using `cv2.adaptiveThreshold()`. The threshold for pixel **X** is the median of Gaussian weighed brightness values of all pixels of the neighbourhood (red square, defined by `blocksize`). From that value, the constant `C` is substracted. If the actual brightness value is above the corrected threshold, the according pixel of the output image gets a white representative (black otherwise).

S.2 Effect of top-hat algorithm

Figure S2 shows the effect of the *top-hat* algorithm. Small objects near to a bigger and shiny object can be detected, which would be overlooked otherwise.

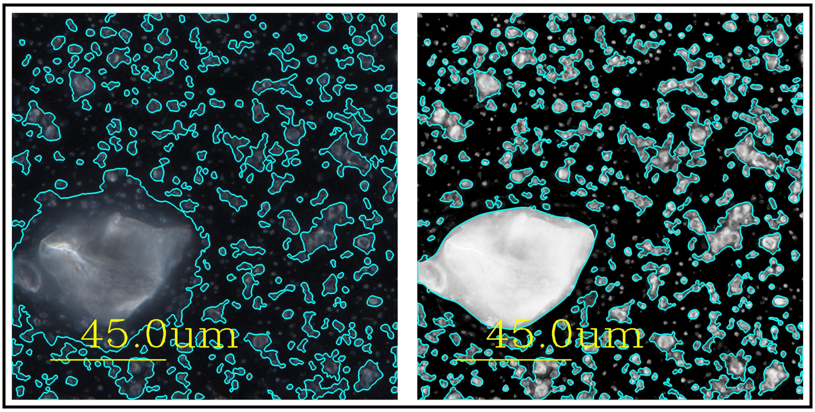

Fig. S2 Illustration of the effect of the *top-hat* algorithm (right) being applied on a typical image of fragments deposited on the filter surface.

S.3 Single results of repetitive measurements on reference particles

The evolution of bootstrap based confidence intervals are shown for the ten repetitive measurements on reference particles (PET, PS, PLA) [2]. The results are evaluated in section **??** (figure S3 to S12).

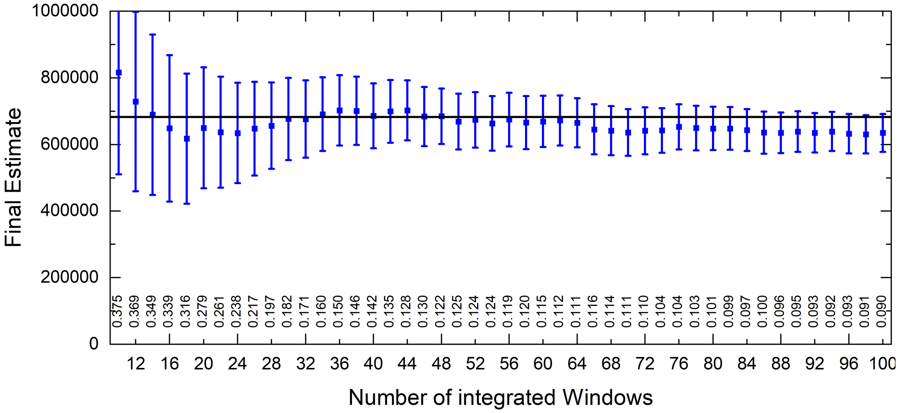

Fig. S3 Repetitive measurements on reference particles (section **??**). Result of the first measurement (evolution of the estimated confidence interval).

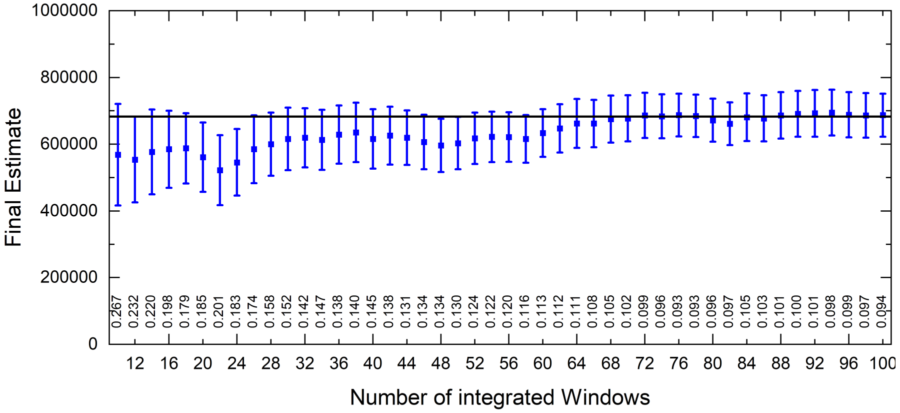

Fig. S4 Result of the second measurement.

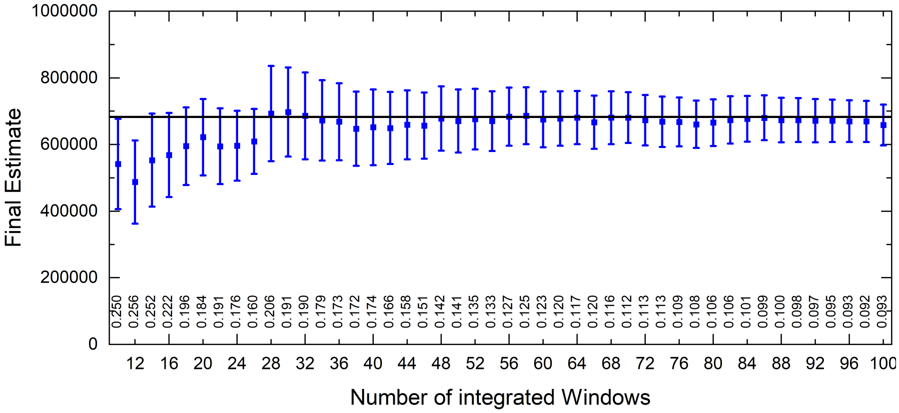

Fig. S5 Result of the third measurement.

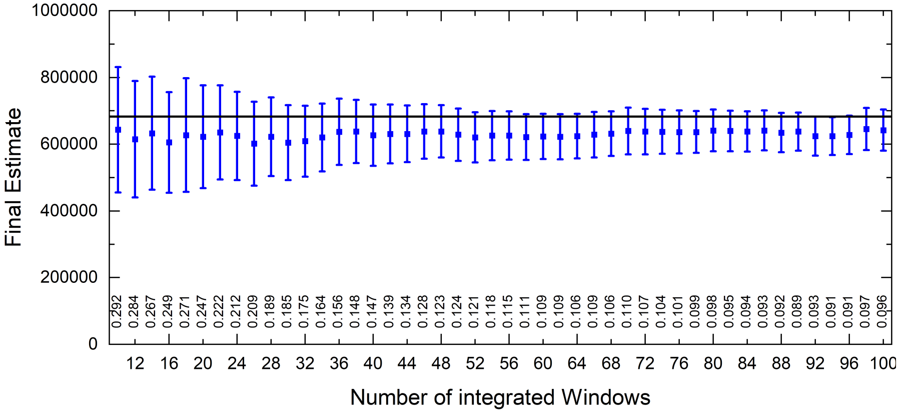

Fig. S6 Result of the fourth measurement.

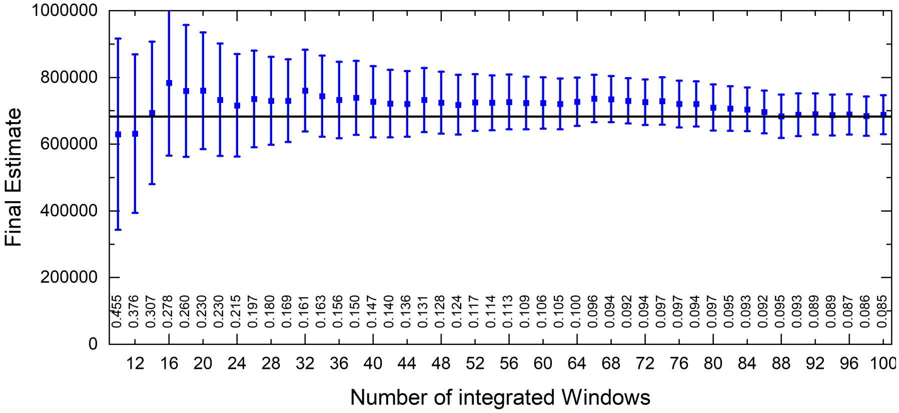

Fig. S7 Result of the fifth measurement.

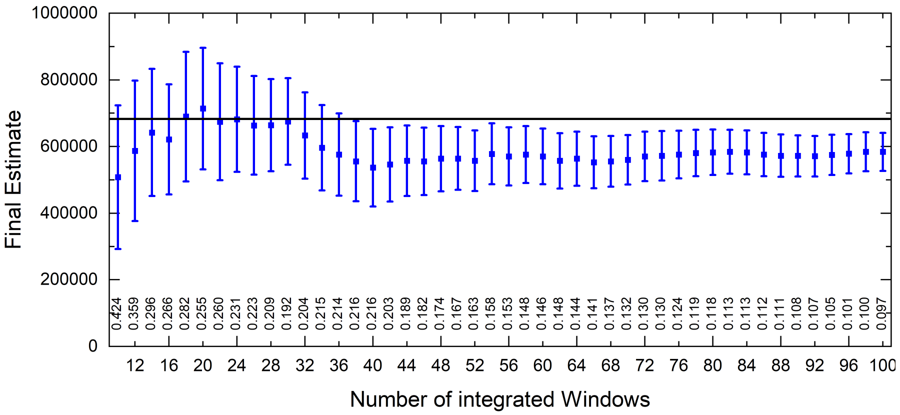

Fig. S8 Result of the sixt measurement.

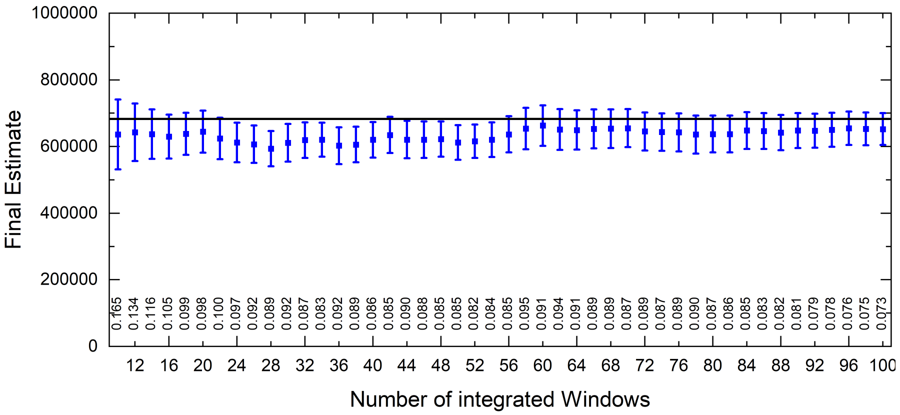

Fig. S9 Result of the seventh measurement.

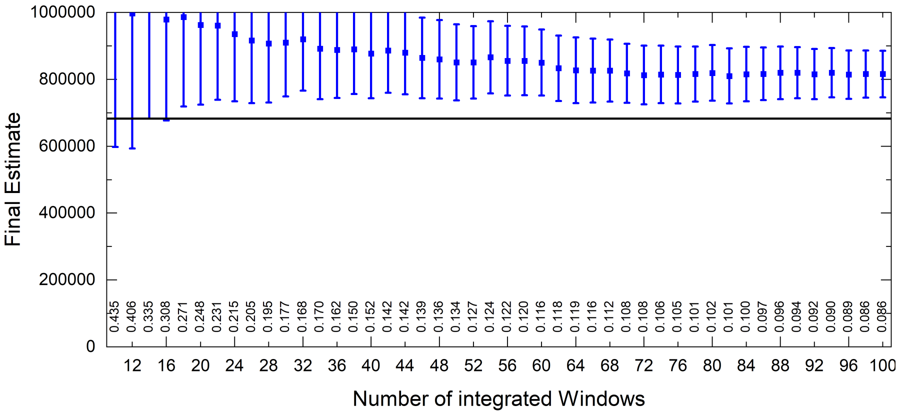

Fig. S10 Result of the eighth measurement.

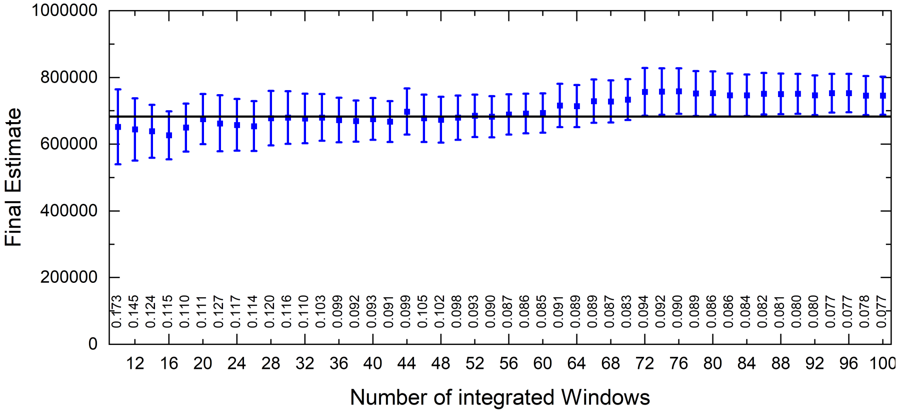

Fig. S11 Result of the ninth measurement.

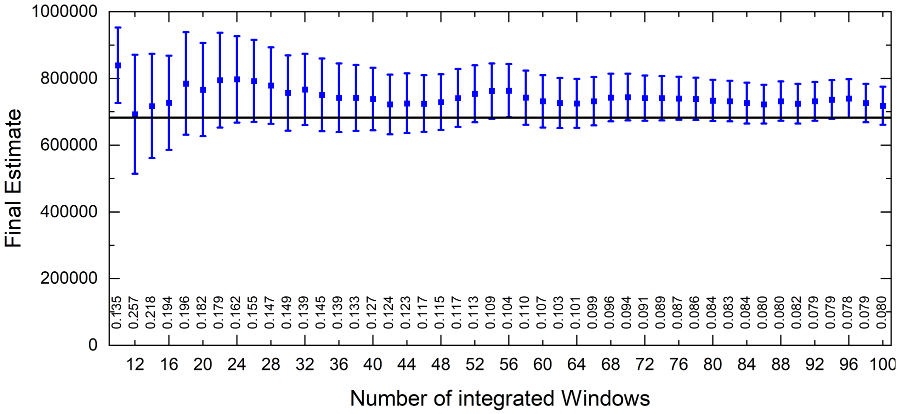

Fig. S12 Result of the tenth measurement.

References

[1] E. von der Esch, A.J. Kohles, P.M. Anger, R. Hoppe, R. Niessner, M. Elsner, N.P. Ivleva, Tum-particletyper: A detection and quantification tool for automated analysis of (microplastic) particles and fibers. PLOS One **15**(6), e0234,766 (2020). https://doi.org/10.1371/journal.pone.0234766

[2] E. von der Esch, M. Lanzinger, A.J. Kohles, C. Schwaferts, J. Weisser, T. Hofmann, K. Glas, M. Elsner, N.P. Ivleva, Simple generation of suspensible secondary microplastic reference particles via ultrasound treatment. Front. Chem. **8**, 169 (2020). https://doi.org/10.3389/fchem.2020.00169
